# Carrier-mediated ferromagnetism in two-dimensional PtS_2_†

**DOI:** 10.1039/c9ra09756b

**Published:** 2020-01-03

**Authors:** Konstantina Iordanidou, Michel Houssa, Clas Persson

**Affiliations:** Centre for Materials Science and Nanotechnology, Department of Physics, University of Oslo P.O. Box 1048 Blindern NO-0316 Oslo Norway konstantina.iordanidou@smn.uio.no; Department of Physics and Astronomy, University of Leuven B-3001 Leuven Belgium

## Abstract

Using first principles calculations based on density functional theory, we study the impact of hole doping on the magnetic and electronic properties of two dimensional PtS_2_. Although 2D PtS_2_ is intrinsically non-magnetic, a stable ferromagnetic phase is found for a wide range of hole densities, owing to the so-called Stoner instabilities. Besides spontaneous magnetization, half-metallicity is additionally observed. The majority and minority spin states exhibit insulating and metallic nature, respectively, allowing a fully polarized spin transport in 2D PtS_2_. Lastly, hole doping resulting from substitutional doping is investigated. For As-doped PtS_2_ shallow spin-polarized states close to the valence band edge are observed, and among all studied group-V dopants, As replacing S, is the most promising one to induce p-type conductivity and a subsequent ferromagnetic order in PtS_2_.

## Abstract 2

Using first principles calculations based on density functional theory the impact of hole doping on the magnetic and electronic properties of two dimensional PtS_2_ is studied.

## Graphical abstract

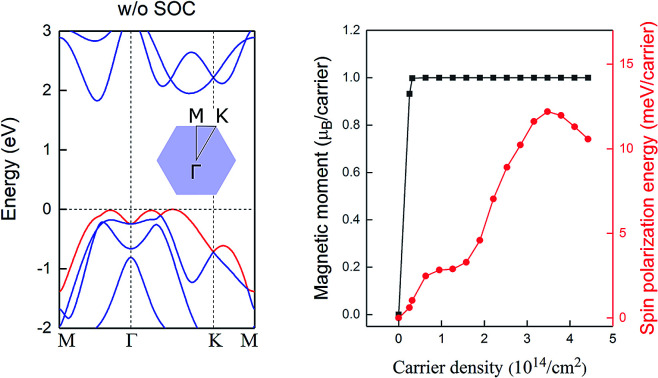

## 1. Introduction

Over the past decades, the technological advances yielded to smaller transistors and subsequently to devices integrating an increasing number of them. However, as the physical dimensions of the transistors are scaled down, quantum effects start to dominate the device operation. In particular, transistors with very short channel lengths experience source to drain tunneling currents, *i.e.*, high off-state currents which increase the static power consumption and cause heat dissipation. To overcome such limitations, new materials that can sustain aggressive scaling and/or new technologies should be introduced. The “marriage” of two dimensional materials with spintronics^1–3^ may bring a revolution in today's transistor technology.

To realize spintronic nanodevices the selection of materials is critical. For instance, the spin states should be easily established and conveniently manipulated, whereas long-distance spin transport and efficient spin injection should be achieved. Although various spintronic nanodevices have been realized in the last years, the quest for optimal materials is still an open issue. Remarkably, recent experimental observations revealed a long-range ferromagnetic order in 2D compounds like CrI_3_ and Cr_2_Ge_2_Te_6_.^4,5^ In addition, spontaneous magnetization has been predicted in monolayer metal chalcogenides, such as GaS, GaSe and InSe, upon hole doping.^6–9^ Such magnetization arises from an exchange splitting of the electronic states at the valence band edge, where the density of states exhibits a sharp van Hove singularity, leading to the so-called Stoner instability.

Two dimensional PtS_2_ is a relatively unexplored member of the transition metal dichalcogenide (TMD) family. Its thermodynamically stable structure consists of one hexagonal metal plane sandwiched between two hexagonal chalcogen planes with an octahedral coordination, forming the so-called 1T phase.^10,11^ Interestingly, a high mobility of 1107 cm^2^ V^−1^ s^−1^ is theoretically predicted for 2D PtS_2_, which is larger compared to other well-studied TMDs like MoS_2_.^12^ In addition, theoretical investigations reveal a transition from a non-magnetic to a ferromagnetic phase upon hydrogenation, and the magnetic moments mainly arise from the Pt 5d orbitals.^13^

In this paper, the magnetic and electronic properties of hole-doped 2D PtS_2_ are studied, using first principles calculations based on density functional theory (DFT). Although 2D PtS_2_ is intrinsically non-magnetic, a stable ferromagnetic phase is found for a wide range of hole densities. Hole doping resulting from substitutional doping is also investigated.

## 2. Models and computational methods

First principles calculations of monolayer PtS_2_ are performed using the spin polarized DFT, as implemented in the Vienna *ab initio* simulation package (VASP).^14,15^ The generalized gradient approximation, developed by Perdew, Burke and Ernzerhof (PBE), is used for the exchange correlation functional,^16^ and long-range van der Waals corrections are applied, using the DFT-D3 Grimme method.^17^ The projected augmented wave (PAW) pseudopotentials are employed,^18^ along with a kinetic energy cutoff of 400 eV. The Brillouin zone is sampled by a 4 × 4 × 1 *k*-point grid for the atomic relaxations, whereas a denser grid is considered for the electronic structure calculations. Note that in order to get well-converged magnetic moments, a grid of 18 × 18 × 1 *k*-points is adopted. The atomic positions are optimized using the conjugate gradient method, with 10^−2^ eV Å^−1^ force convergence criteria on the ionic optimization. For our calculations we used the tetrahedron method with Bloch's corrections whereas for the calculations of the total magnetic moments of hole-doped systems, different smearing methods were tested, like the Methfessel–Paxton method and the Gaussian smearing, leading to identical results.

We additionally performed hybrid functional calculations, using the VASP code. The functional proposed by Heyd, Scuseria and Ernzerhof (HSE) was adopted,^19^ along with the standard mixing (25% Fock exchange) and range-separation parameters. Note that for the HSE calculations, we used a less dense *k*-point grid as compared to the PBE calculations.

We constructed 5 × 5 single-layer supercells consisting of one hexagonal Pt plane sandwiched between two hexagonal S planes, with ∼17.8 × 17.8 Å lateral dimensions and 75 atoms in total. Periodic boundary conditions were applied, and a vacuum layer larger than 15 Å was used in the out-of-plane direction, to avoid any artificial interactions between periodic images. Hole doping was achieved by changing the number of electrons of the supercell and by adding a compensating jellium background of opposite charge.

## 3. Results and discussion

### 3.1. Ferromagnetism in hole-doped 2D PtS_2_

**Fig. 1:**
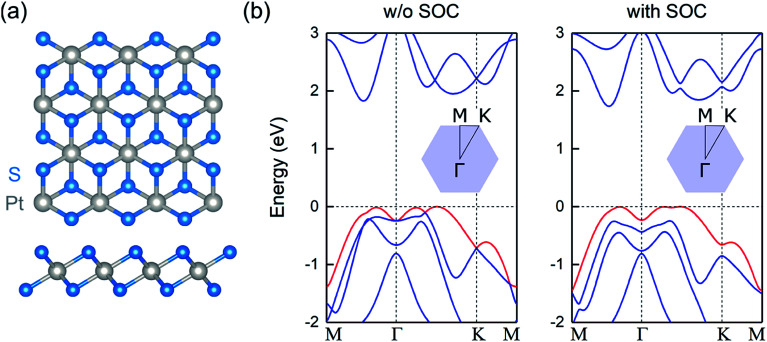
(a) Top and side views of the atomic structure of monolayer PtS_2_, where grey and blue spheres correspond to Pt and S atoms, respectively (b) electronic band structure of monolayer PtS_2_ without SOC (left panel) and with SOC (right panel). The energies refer to the VB maximum and the topmost VB is highlighted in red. The insets show the corresponding Brillouin zone.

The relaxed atomic structure of monolayer PtS_2_ is shown in Fig. 1a. For the optimized unit cell, the in-plane lattice constant is 3.56 Å, in excellent agreement with experimental observations.^20^ The S–Pt bond length, S–Pt–S bond angle, and S–S vertical distance are found to be 2.40 Å, 84.1°, and 2.47 Å, respectively.

As observed in Fig. 1b, single-layer PtS_2_ is a semiconductor with an indirect band gap of *E*_g_ = 1.83 eV. The valence band (VB) maximum lies along the Γ–K direction, whereas the conduction band (CB) minimum is located between the Γ and M points, in agreement with previously reported theoretical calculations.^21^ The inclusion of spin–orbit coupling (SOC) reduces the gap by less than 0.1 eV, and retains the indirect nature of the gap. Interestingly, the topmost VB resembles an inverted Mexican-hat-like dispersion. This peculiar VB dispersion leads to a divergence in the density of states, corresponding to sharp van Hove singularities. When the Fermi level lies close to such singularities, the large electronic density of states favors the formation of a ferromagnetic order, since the Stoner criterion is satisfied.^22–24^ Thus, hole-doped 2D PtS_2_ can be a ferromagnetic material, similar to other 2D metal chalcogenides.^6–8^ Note that the inclusion of SOC preserves the sombrero shape VB and the subsequent van Hove singularity at the top of the VB and for the calculations of the magnetic and electronic properties of doped systems, this relativistic interaction is not included.

**Fig. 2:**
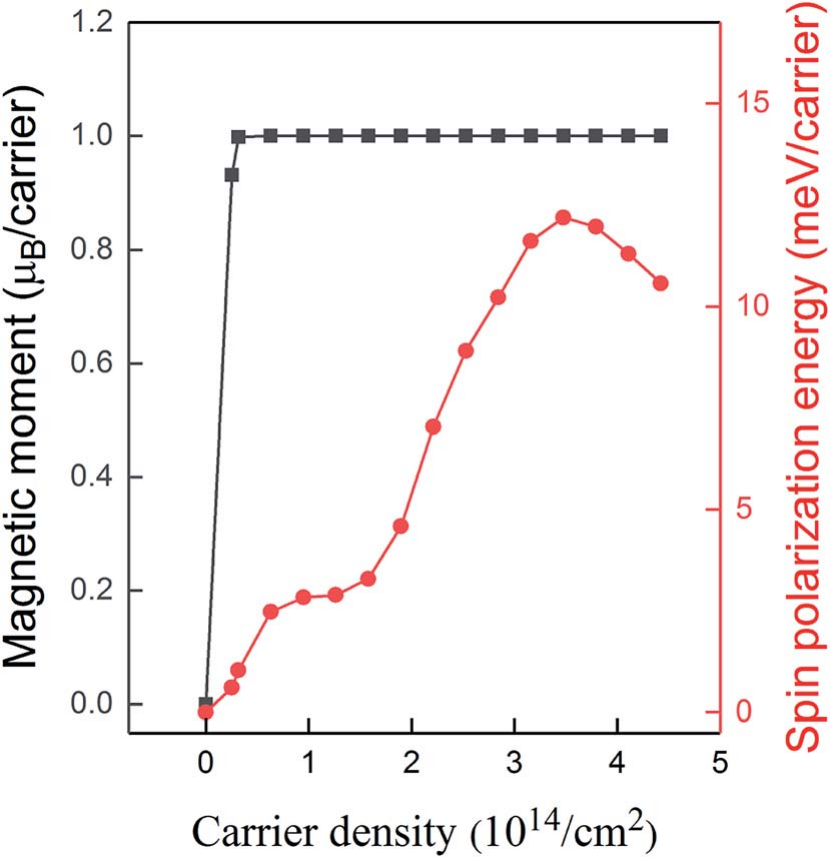
Magnetic moment and spin polarization energy as a function of the carrier density.

Initially, we consider charged supercells and we compute the magnetic moment and the spin polarization energy for different hole densities (*n*). The spin polarization energy is defined as the energy difference between the non-magnetic and the ferromagnetic phase, and positive values reveal a stable ferromagnetic phase. As observed in Fig. 2, at the low doping level, a ferromagnetic state spontaneously emerges and the magnetic moment reaches the saturation value of 1 *μ*_B_ per carrier. Although the magnetic moment per carrier remains constant up to high hole densities, the spin polarization energy per carrier significantly varies. In particular, for low hole densities, it increases by increasing the density, whereas for *n* = 3.5 × 10^14^/cm^2^, it reaches the maximum value of about 12 meV per carrier. Further increase of the hole density results in the reduction of the spin polarization energy.

It is worth noting that for charged slabs, the electrostatic potential significantly diverges with the distance from the slab, and the total energy strongly depends on the vacuum thickness. However, by increasing the vacuum thickness, even though the energy significantly varies, the energy difference between the non-magnetic and the ferromagnetic state remains unchanged.

**Fig. 3:**
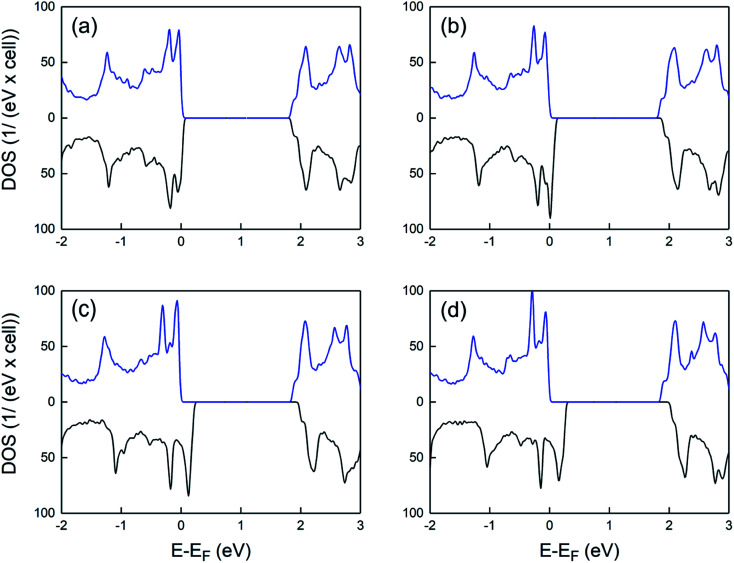
Density of states of charged supercells for different hole densities: (a) 0.6 × 10^14^/cm^2^ (b) 1.9 × 10^14^/cm^2^ (c) 3.8 × 10^14^/cm^2^ and (d) 4.4 × 10^14^/cm^2^. The spectra include a 0.02 eV Lorentzian broadening. Blue and black lines indicate spin-up-like and spin-down-like density of states, respectively.

Fig. 3 shows the spin-polarized density of states of charged supercells at various hole densities. At the low doping level, a slight splitting of the opposite spin-states near the Fermi level is observed. By increasing the hole density, the energy difference between the spin up and spin down VB maxima significantly increases, whereas the Fermi level is shifted to lower energies. This results in a half-metallic behavior where the majority and minority spin states exhibit insulating and metallic nature, respectively. Such behavior is highly promising for future spintronic applications, allowing a fully polarized spin transport in 2D PtS_2_.

In order to verify that the conclusion for the hole doping induced ferromagnetism is not affected by the underestimation of the band gap within DFT, we additionally performed hybrid functional calculations. First, we computed the HSE band structure of pristine 2D PtS_2_ and we found a gap opening of about 0.8 eV. As a next step, the electronic and magnetic properties of hole-doped systems were computed. Similar to our PBE calculations, upon hole doping, a splitting of the opposite spin states near the Fermi level was observed (see ESI, Fig. S1†) and the magnetic moments were found to be 1 *μ*_B_ per carrier.

### 3.2 Hole doping induced by S substitution

Next, we consider 5 × 5 × 1 supercells and we study the substitution of S atoms by group-V atoms, namely X = N, P, and As. Taking into account that S atoms have six valence electrons whereas group-V atoms have only five electrons, such substitutional dopants are good candidates to induce hole doping and a subsequent ferromagnetic order in monolayer PtS_2_. Regarding the structural properties, Pt atoms neighboring the N and P atoms exhibit an inward relaxation of about 12.7 and 3.1%, resulting in shortened N–Pt and P–Pt bonds of 2.09 and 2.32 Å, respectively. By contrast, Pt atoms surrounding the rather large As anion relax outwards by about 1.9%, leading to elongated As–Pt bonds of 2.44 Å.

To evaluate the thermodynamic stability of these dopants, we calculate the dopant formation energies using the equation

1 *E*_for_ = *E*_tot_(PtS_2_:X) − *E*_tot_(PtS_2_) + *μ*_S_ − *μ*_X_

where *E*_tot_(PtS_2_:X) and *E*_tot_(PtS_2_) are the total energies of the doped and pristine systems, respectively, whereas *μ*_S_ and *μ*_X_ are the chemical potentials of sulfur and dopant atoms, respectively. Note that the sulfur chemical potential refers to the solid structure of sulfur, *i.e.*, *μ*_S_ = *μ*^sol^_S_ + Δ*μ*_S_, and the dopant chemical potential corresponds to solid phosphorus and arsenic, but molecular nitrogen. The N, P, and As formation energies are found to be 3.1 + Δ*μ*_S_, 0.9 + Δ*μ*_S_, and 1.1 + Δ*μ*_S_ eV, respectively, *i.e.*, P and As have fairly similar formation energies whereas the N formation energy is significantly larger, which is consistent with the larger lattice distortion.

**Fig. 4:**
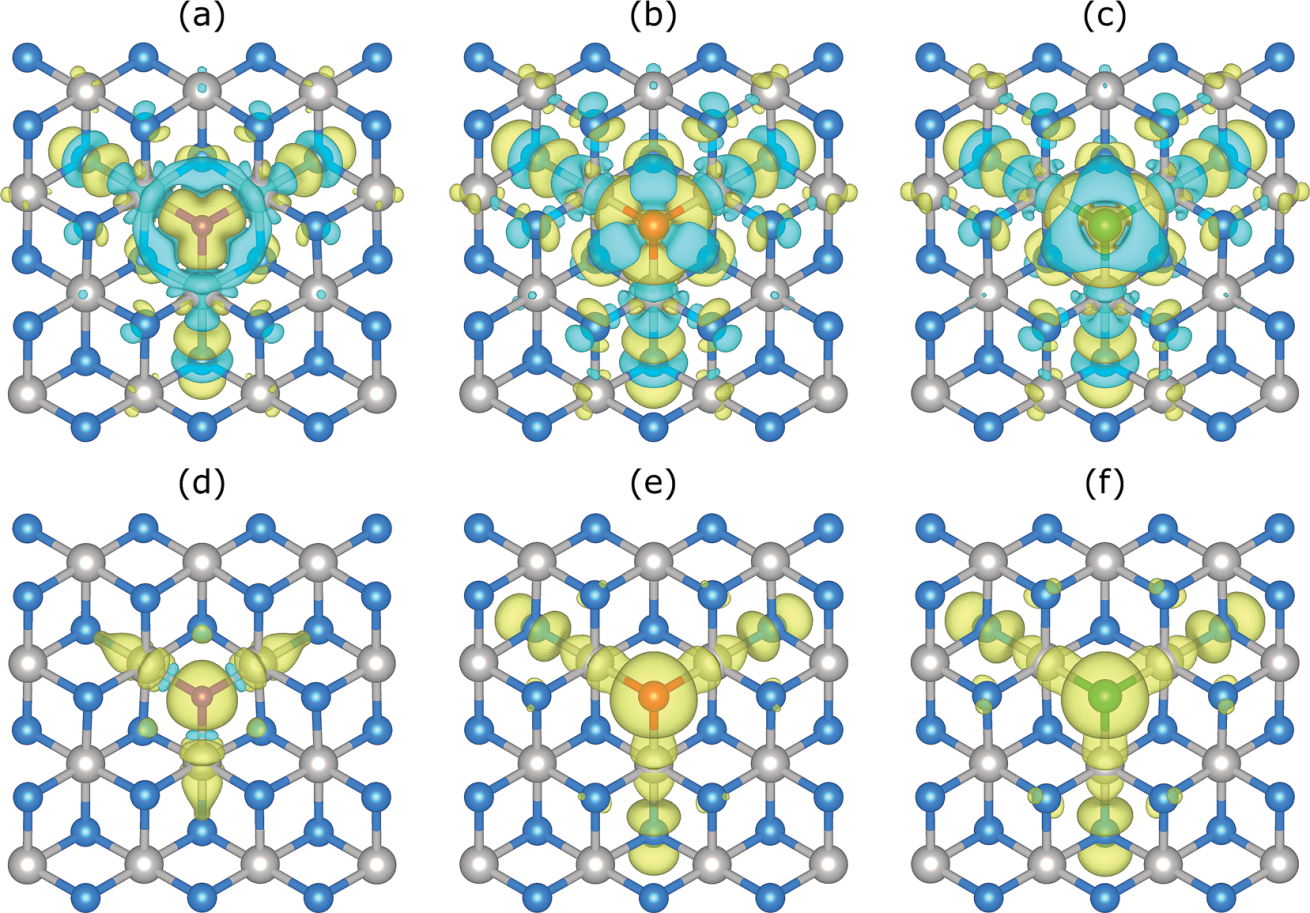
Charge density differences Δ*ρ*_c_ = *ρ*_PtS_2_:X_ − *ρ*_PtS_2_:V_S__ − *ρ*_X_ (top panels) and spin density differences Δ*ρ*_s_ = *ρ*_↑_ − *ρ*_↓_ (bottom panels) of group-V doped systems. (a) and (d) N-on-S site, (b) and (e) P-on-S site, (c) and (f) As-on-S site. Purple, red and green spheres correspond to N, P, and As atoms, respectively. The isosurface value is 0.001 electrons per Å^3^. For the charge density differences, yellow and blue isosurfaces refer to electron accumulation and depletion, respectively.

Next, the magnetic and electronic properties of doped systems are discussed. All substitutional dopants lead to magnetic ground states with total magnetic moments of 1 *μ*_B_. The spin polarization energies are found to be 0.27, 0.10, and 0.06 eV, for N, P, and As, respectively, where the positive values reveal a stable (local) magnetic moment. The charge density differences as well as the spin density differences for all doped systems are shown in Fig. 4. The charge density difference is computed by Δ*ρ*_c_ = *ρ*_PtS_2_:X_ − *ρ*_PtS_2_:V_S__ − *ρ*_X_ where *ρ*_PtS_2_:X_, *ρ*_PtS_2_:V_S__, and *ρ*_X_ are the charge densities of doped PtS_2_ (*i.e.* PtS_2_ with a S substituent), bare PtS_2_:V_S_ (*i.e.* PtS_2_ with a S vacancy), and isolated X dopants, respectively. Accordingly, the spin density difference, which is used to visualize the magnetic moment distribution around the dopant atom, is computed by Δ*ρ*_s_ = *ρ*_↑_ − *ρ*_↓_ where *ρ*_↑_ and *ρ*_↓_ are the spin up and spin down charge densities, respectively. To further describe the magnetic moment distribution, the ratio between the magnetic moment of the dopant atom and the total magnetic moment (*M*_r,X_ = *M*_X_/*M*_tot_) is computed. We find *M*_r,N_ ≈ 66%, *M*_r,P_ ≈ 32%, and *M*_r,As_ ≈ 26%, *i.e.*, the magnetic moment is less localized in the P and As doped systems, compared to the N doped one, as expected from the much deeper in-gap states for N doping.

**Fig. 5:**
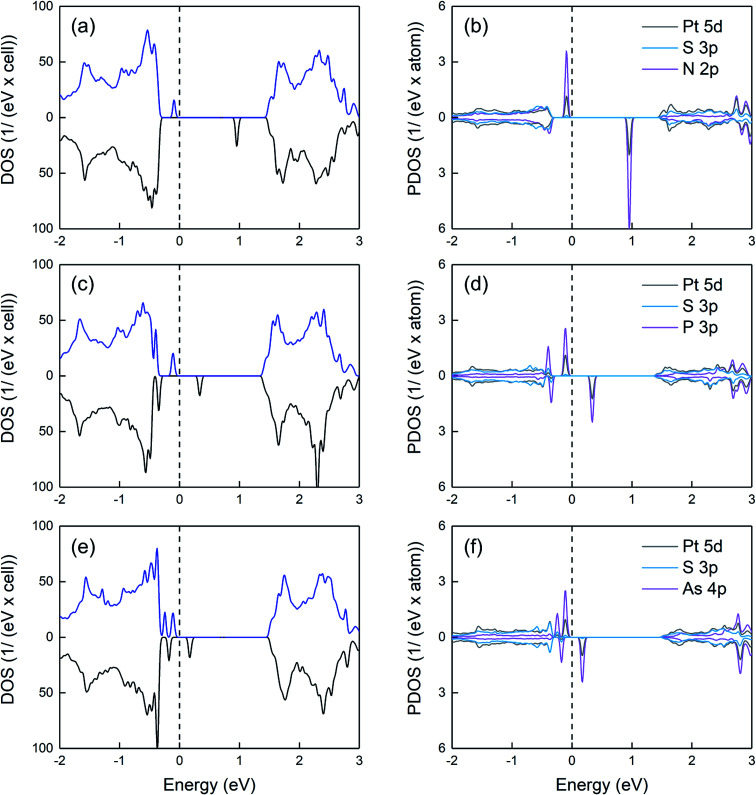
Density of states (left panels) and projected density of states (right panels) of group-V doped systems (a) and (b) N-on-S site, (c) and (d) P-on-S site, (e) and (f) As-on-S site. For the projected density of states, Pt and S neighboring the dopant atom are considered. The energies refer to the highest occupied state and the spectra include a 0.02 eV Lorentzian broadening.

Regarding the electronic properties, as observed in Fig. 5, N creates two spin-polarized states within the gap. The occupied spin-up-like and unoccupied spin-down-like states are located ∼ 0.2 and 1.3 eV above the VB edge. Taking into account that the empty state lies far away from the VB, the substitution of S by N is unlikely to lead to a p-type behavior. Similar to N, both P and As dopants produce spin polarized states within the gap. By increasing the dopant atomic number, the gap states move towards the valence band and their spin splitting reduces. Note that the dependence of the spin splitting on the atomic number follows the trend for the exchange correlation integral.^25,26^ Overall, among all three dopants, As is the most promising one to induce p-type conductivity (provided a sufficient dopant concentration can be achieved), since the unoccupied gap state lies close to the VB edge. Our projected density of states calculations reveal that for all doped systems, the opposite spin states within the gap are mainly derived from the group-V p orbitals as well as the 5d orbitals of Pt atoms neighboring the group-V atom.

## 4. Conclusions

Based on first principles calculations, we studied the magnetic and electronic properties of hole-doped monolayer PtS_2_. Upon hole doping, a transition from a non-magnetic to a ferromagnetic phase along with a half-metallic behavior were observed. Therefore, hole-doped 2D PtS_2_ presenting one conducting and one insulating spin channel, could be highly promising for future spin-based nanodevices. Hole doping resulting from substitutional doping was also investigated. We found that group-V atoms replacing S atoms create spin polarized states within the gap. By increasing the dopant atomic number, these states move towards the valence band edge and their spin splitting reduces, potentially forming a ferromagnetic phase in monolayer PtS_2_.

## Conflicts of interest

There are no conflicts to declare.

## Supplementary Material

RA-010-C9RA09756B-s001
