# Supplementary material for: Carrier-mediated ferromagnetism in two-dimensional PtS2
Source: RSC Adv. 2020 Jan 3;10(2):952–7. doi: 10.1039/c9ra09756b (PMC9047313; doi:10.1039/c9ra09756b)
Supplement: RA-010-C9RA09756B-s001 [file RA-010-C9RA09756B-s001.pdf]

Electronic Supplementary Material (ESI) for RSC Advances.
This journal is © The Royal Society of Chemistry 2020

Supplementary Information

Carrier-mediated ferromagnetism in two-dimensional PtS_2

Konstantina Iordanidou[1], Michel Houssa[2], Clas Persson[1]

[1] Centre for Materials Science and Nanotechnology, Department of Physics, University of Oslo, P.O. Box 1048 Blindern, NO-0316 Oslo, Norway

[2]Department of Physics and Astronomy, University of Leuven, B-3001 Leuven, Belgium

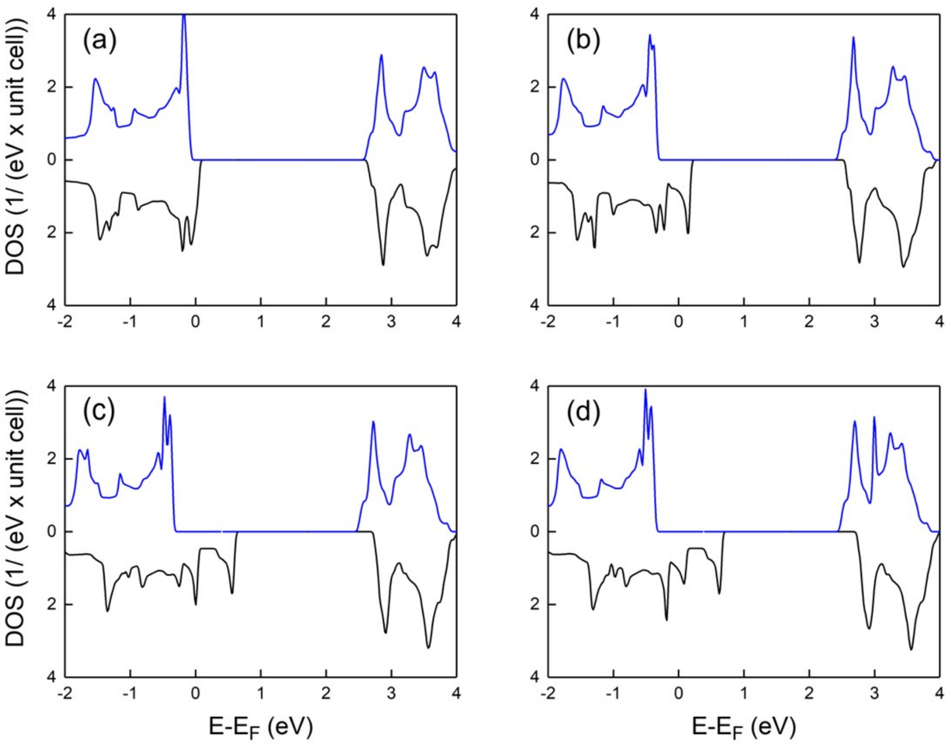

Figure S1: Density of states of charged unit cells for different hole densities: (a) $0.6 \times 10^{14}/cm^2$, (b) $1.9 \times 10^{14}/cm^2$, (c) $3.8 \times 10^{14}/cm^2$, and (d) $4.4 \times 10^{14}/cm^2$, using the HSE functional. The spectra include a 0.02 eV Lorentzian broadening. Blue and black lines indicate spin-up-like and spin-down-like density of states, respectively.
